# Elotuzumab: The First Monoclonal Antibody for the Treatment of Multiple Myeloma

**DOI:** 10.6004/jadpro.2016.7.5.6

**Published:** 2016-07-01

**Authors:** Karen M. Fancher,(1,2), Elizabeth J. Bunk,(1)

**Affiliations:** 1Duquesne University Mylan School of Pharmacy, Pittsburgh, Pennsylvania; 2University of Pittsburgh Medical Center Passavant, Pittsburgh, Pennsylvania

Multiple myeloma (MM) is a hematologic cancer in which malignant plasma cells are formed. Plasma cells are mainly found in bone marrow and function within the body to attack and destroy antigens. Multiple myeloma occurs when malignant plasma cells accumulate in the bone marrow, leading to marrow failure and destruction of the bone.

As the second most commonly diagnosed hematologic malignancy in the United States, 30,330 new cases of MM are estimated in 2016 (American Cancer Society, 2016). Although approximately 45% of patients survive 5 years after diagnosis, the disease remains incurable (Ashjian & Redic, 2015). In the past decade, there have been a number of recent therapeutic advances to address the current limitations.

Initial treatment of MM varies depending on age, stage at diagnosis, and patient-specific factors such as comorbid conditions and cytogenetics. Current National Comprehensive Cancer Network (NCCN) guidelines for newly diagnosed MM involve two or more drugs in combination (NCCN, 2016). Although there are many treatment options available, multiple myeloma has a very high relapsing and refractory nature, and thus there is a need for further research and development of novel drugs in this setting. With the advent of innovative treatments with different mechanisms of action targeting specific proteins, agents such as elotuzumab (Empliciti) create an option for these relapsed patients.

## MECHANISM OF ACTION

Elotuzumab is a first-in-class humanized monoclonal antibody that is targeted against a glycoprotein known as cell-surface glycoprotein CD2 subset 1 (CS1, also known as signaling lymphocyte activation molecule F7, or SLAMF7; Lonial et al., 2015; Magarotto, Salvini, Bonello, Bringhen, & Palumbo, 2016). The glycoprotein CS1 mediates the adhesion of multiple myeloma cells to the bone marrow stromal cells, leading to their proliferation and survival (Magarotto et al., 2016). It is expressed on 95% of bone marrow myeloma and a portion of natural killer cells but not on normal hematopoietic stem cells, other lymphocytes, epithelia, vessels, or smooth muscle cells (Ashjian & Redic, 2015; Lonial et al., 2015; Magarotto et al., 2016). The expression of CS1 is unique in that it is independent of treatment history, chromosomal abnormalities, molecular subtypes, or disease risk, suggesting this target may be useful even in patients with poor prognostic markers or after multiple lines of drug therapy (Liu, Szmania, & van Rhee, 2014; Lonial et al., 2016).

The precise mechanism of action of elotuzumab is not fully elucidated, but it is postulated that it directly activates natural killer cells through the CS1 pathway. It also targets CS1 on myeloma cells and facilitates the interaction with natural killer cells to mediate the killing of myeloma cells through antibody-dependent cellular cytotoxicity (ADCC; Bristol-Myers Squibb, 2015).

## CLINICAL STUDIES

**Phase I Studies**

An early phase I dose-escalation study in patients with advanced MM evaluated elotuzumab at six dose levels, ranging from 0.5 mg/kg to 20 mg/kg. In the original protocol, the administration of premedications to prevent infusion-related reactions was optional. After the observation of infusion-related events, the study was amended to require premedications prior to drug administration. Results showed no objective responses, demonstrating disappointing activity when elotuzumab was given as a single agent (Zonder et al., 2012).

More promising results were seen in a phase I study in relapsed and refractory MM, where elotuzumab at 5, 10, or 20 mg/kg intravenously (IV) was used in combination with dexamethasone and lenalidomide (Revlimid). The combination regimen resulted in an objective response rate of 82% of treated patients (Lonial et al., 2012).

**Phase II Studies**

A phase II study evaluating the safety and efficacy of elotuzumab in patients with confirmed relapsed MM compared a 10-mg/kg dosing regimen with a 20-mg/kg dosing regimen, both in combination with lenalidomide and dexamethasone. Premedications to prevent infusion-related reactions were required in this study based on phase I observations.

The objective response rate in the 10-mg/kg group was 92%, whereas the 20-mg/kg group had a 76% response rate. The most common treatment-related adverse effects seen were diarrhea, constipation, muscle spasms, back pain, nausea, fatigue, and upper respiratory infections, with the most common grade 3/4 events being lymphopenia and neutropenia (Richardson et al., 2015).

**Phase III Trials**

Two phase III clinical trials, ELOQUENT-1 and ELOQUENT-2, are evaluating the efficacy and safety of elotuzumab in combination with lenalidomide and dexamethasone compared with lenalidomide and dexamethasone alone. ELOQUENT-2 has been completed, and study results have been published, whereas ELOQUENT-1 has not yet completed accrual. In late 2015, elotuzumab was approved by the US Food and Drug Administration (FDA) for use in combination with lenalidomide and dexamethasone for the treatment of patients with MM who have received one to three prior therapies based on the results of the ELOQUENT-2 trial.

*ELOQUENT-1*: This is an open-label trial of lenalidomide and dexamethasone with or without elotuzumab in patients with previously untreated MM. Participants are newly diagnosed and must not have received any prior systemic antimyeloma therapy, have measurable disease, and not be candidates for high-dose therapy plus stem cell transplantation. The primary outcome measure is progression-free survival (PFS), defined as the time from randomization to the date of first tumor progression or death. This study is ongoing but no longer recruiting participants. The estimated final data collection date for the primary outcome is April 2018 (ClinicalTrials.gov, 2016).

*ELOQUENT-2*: The results of this trial were recently published (Lonial et al., 2015). This was an open-label, randomized, multicenter trial of 646 MM patients who had received one to three previous therapies and had documented progression of disease. Primary endpoints included PFS and overall response rate.

Patients were randomly assigned to receive either elotuzumab at 10 mg/kg with lenalidomide and dexamethasone (n = 321) or lenalidomide and dexamethasone alone (n = 325). Randomization was stratified according to baseline macroglobulin level, number of previous therapies, and previous immunomodulatory drugs (Lonial et al., 2015). Baseline characteristics were balanced between the two groups. A maximum of 10% of participants who had received previous lenalidomide could enroll in the study.

At 1 year, the rate of PFS in the elotuzumab group was 68% vs. 57% in the control group. At 2 years, the PFS rates were 41% and 27%, respectively. Median PFS in the elotuzumab group was 19.4 months (95% confidence interval [CI], 16.6–22.2 months) vs. 14.9 months (95% CI, 12.1–17.2 months) in the control group, with a hazard ratio of 0.70 (95% CI, 0.57–0.85, *p* < .001). The benefit of adding elotuzumab was observed across most subgroups, including patients with resistance to previous therapy; those who had treatment with previous immunomodulatory drugs including bortezomib (Velcade); or patients with high-risk cytogenetic profiles, particularly del(17p). The overall response rate was 79% in the elotuzumab group vs. 66% in the control group (*p* < .001; Lonial et al., 2015).

These results show that the addition of elotuzumab to lenalidomide and dexamethasone provided a statistically significant improvement in both disease-free survival and treatment outcomes. Study investigators reported that this combination of therapy resulted in a relative reduction of 30% in the risk of disease progression or death (Lonial et al., 2015). Based on the reported results of this study, elotuzumab received FDA approval in November 2015. The final analysis of the ELOQUENT-2 study will be completed in March 2018 (ClinicalTrials.gov, 2016).

**Ongoing Research and Future Studies**

A phase I/II study is ongoing to evaluate the addition of elotuzumab to lenalidomide, bortezomib, and dexamethasone in previously untreated high-risk MM patients. As described previously, ELOQUENT-1 is also an ongoing trial, with pending results. There are also several studies that are recruiting both relapsed and newly diagnosed MM patients for various combinations of elotuzumab, lenalidomide, dexamethasone, and bortezomib (Markham, 2016). Future research involves evaluating elotuzumb in combination with other agents in both relapsed and refractory disease as well as newly diagnosed MM.

## DOSING AND ADMINISTRATION

At this time, elotuzumab is exclusively indicated in combination with lenalidomide and dexamethasone in patients with MM who have received one to three prior therapies (Bristol-Myers Squibb, 2015).

The recommended dose of elotuzumab is 10 mg/kg IV every week for cycles 1 and 2 and then every 2 weeks thereafter. Premedication with dexamethasone, acetaminophen, diphenhydramine, and ranitidine (or equivalent agents) is required as per the prescribing information. The infusion should be initiated at a rate of 0.5 mL/min and may be increased in a stepwise fashion every 30 minutes if infusion reactions do not occur (Bristol-Myers Squibb, 2015).

**Adverse Effects**

The primary adverse effects noted in phase I and II trials were infusion reactions, including pyrexia, chills, hypertension, nausea, and/or vomiting (Liu et al., 2014; van de Donk et al., 2016). After protocol modifications of early trials to include premedications, these reactions were less frequent and manageable (Liu et al., 2014; Lonial et al., 2015). The phase III ELOQUENT-2 trial reported infusion reactions in 10% of the study population, with 70% of reactions occurring during the first infusion (Lonial et al., 2015).

Other adverse events noted in both arms of the phase III ELOQUENT-2 trial included lymphocytopenia, neutropenia, fatigue, and pneumonia. The incidence of these adverse effects is further illustrated in Table 1. Signs of increased autoimmunity or other effects of immune dysregulation were not noted to be different between the study arms, as the rate of infection and the incidence of opportunistic infections were similar between the groups (Lonial et al., 2015).

**Table 1:**
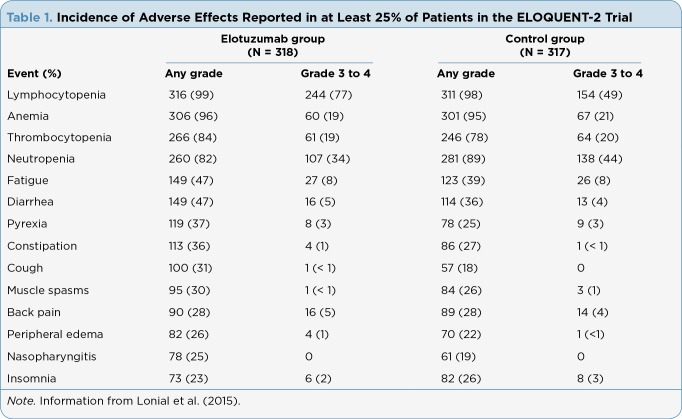
Incidence of Adverse Effects Reported in at Least 25% of Patients in the ELOQUENT-2 Trial

## IMPLICATIONS FOR ADVANCED PRACTITIONERS

Activities of critical importance for advanced practitioners (APs) with regard to elotuzumab include patient and family counseling, monitoring and management of adverse effects, and health-care professional education.

Advanced practitioners should monitor a complete blood cell count prior to each dose of elotuzumab due to the potential for myelosuppression. The risk of myelosuppresion may be augmented by the use of lenalidomide, which is also frequently associated with this complication. There are no specific recommendations for dose adjustments of elotuzumab if myelosuppression occurs; dose delays and modifications of lenalidomide should be considered as per its prescribing information, and clinical judgment may be necessary.

Patients receiving elotuzumab should be frequently monitored for the presence of infection. Serum chemistries should also be routinely examined, since increases in liver function tests were observed in a small percentage of patients in clinical trials of this agent.

The administration of premedications is mandatory to prevent infusion-related reactions. Recommended premedications include dexamethasone, diphenhydramine (or an equivalent histamine-1 blocker), ranitidine (or an equivalent histamine-2 blocker), and acetaminophen. The recommended premedication schedule is somewhat complex, since patients are also receiving concurrent dexamethasone as part of the MM treatment regimen (Bristol-Myers Squibb, 2015). The recommended premedication schema is illustrated in Table 2. Particular attention should be paid to the doses and schedule of premedications.

**Table 2:**
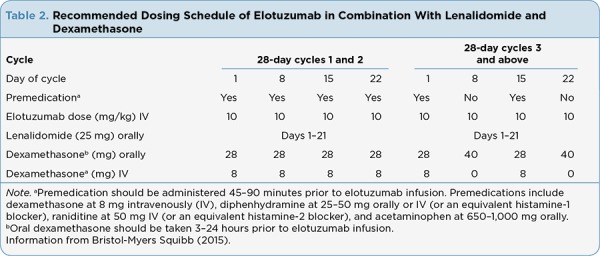
Recommended Dosing Schedule of Elotuzumab in Combination With Lenalidomide and Dexamethasone

During receipt of elotuzumab, patients should be closely observed for symptoms of infusion-related reactions. Infusion reactions typically occur early in the infusion as well as early in the patient’s treatment course. If an infusion-related reaction occurs, the infusion should be interrupted, and appropriate supportive measures should be implemented. When symptoms resolve, the infusion may be restarted according to the description in the prescribing information. The rate of infusion of elotuzumab may be increased according to the schedule in Table 3 if patients do not experience infusion-related reactions (Bristol-Myers Squibb, 2015).

**Table 3:**
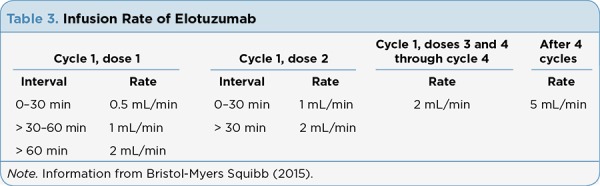
Infusion Rate of Elotuzumab

Practitioners should also be cognizant of the potential adverse effects of lenalidomide and dexamethasone, as these agents will be administered concurrently with elotuzumab therapy.

## SUMMARY

The treatment of relapsed or refractory MM is a significant challenge. Novel treatments that prolong life expectancy while maintaining quality of life are becoming increasingly desirable. The impressive clinical data of elotuzumab coupled with its favorable safety profile make this agent a significant addition to the currently available therapies for MM.
